# LIFE SATISFACTION IN CENTENARIANS: FINDINGS FROM THE SWISS100 MAIN STUDY

**DOI:** 10.1093/geroni/igae098.0216

**Published:** 2024-12-31

**Authors:** Daniela Jopp, Adar Hoffman, Armin von Gunten, Francois Herrmann, Christina Roecke, Mike Martin, Stefano Cavalli

**Affiliations:** University of Lausanne, Lausanne, Vaud, Switzerland; University of Lausanne, Lausanne, Vaud, Switzerland; Lausanne University Hospital, Prilly, Vaud, Switzerland; Geneva University Hospitals, Thônex, Geneve, Switzerland; University of Zurich, Zurich, Zurich, Switzerland; University of Zurich, Zurich, Zurich, Switzerland; University of Applied Sciences and Arts of Southern Switzerland, Manno, Ticino, Switzerland

## Abstract

With increasing numbers of centenarians world-wide, quality of life in these very old individuals has become a topic of high interest, expanding prior biomedical research. Switzerland is not only among the countries with very high life expectancy but is also among the leading countries in terms life satisfaction. In the present study, we investigated the levels and predictors of life satisfaction in Swiss centenarians. The present analysis included a total of 148 centenarians of the SWISS100 Main Study (Mage = 101.8; range: 100-106; 74.3% female). After identification through the national address registry, centenarians were visited for in-person interviews. Centenarians’ health was quite limited: they had an average of six health issues and over half of them lived in care facilities (57.4%). Their cognitive level was moderate to high. Most were widowed, 84% had children, and they reported a substantial number of social contacts. The majority of centenarians (82.4 %) were satisfied with their lives. Correlations indicated that sociodemographic and health variables had only few associations with life satisfaction, but that psychological aspects including optimistic outlook and meaning in life showed notable links. A regression analysis combining significant variables from domain-specific regressions revealed that optimistic outlook was the strongest predictor of interindividual differences in life satisfaction, followed by meaning in life. In sum, findings highlight that Swiss centenarians present poor levels of health yet manage to maintain high life satisfaction through psychological mechanisms, suggesting high resilience in these very old individuals, which replicates and extends results from prior centenarian studies.

